# Electronic Cigarette–Related Contents on Instagram: Observational Study and Exploratory Analysis

**DOI:** 10.2196/21963

**Published:** 2020-11-05

**Authors:** Yankun Gao, Zidian Xie, Li Sun, Chenliang Xu, Dongmei Li

**Affiliations:** 1 University of Rochester Medical Center Rochester, NY United States; 2 University of Rochester Rochester, NY United States

**Keywords:** electronic cigarettes, infodemiology, Instagram, user engagement, exploratory, smoking, e-cig, social media, vape, vaping, risk, public health

## Abstract

**Background:**

Instagram is a popular social networking platform for users to upload pictures sharing their experiences. Instagram has been widely used by vaping companies and stores to promote electronic cigarettes (e-cigarettes), as well as by public health entities to communicate the risks of e-cigarette use (vaping) to the public.

**Objective:**

We aimed to characterize current vaping-related content on Instagram through descriptive analyses.

**Methods:**

From Instagram, 42,951 posts were collected using vaping-related hashtags in November 2019. The posts were grouped as (1) pro-vaping, (2) vaping warning, (3) neutral to vaping, and (4) not related to vaping based on the attitudes to vaping expressed within the posts. From these Instagram posts and the corresponding 18,786 unique Instagram user accounts, 200 pro-vaping and 200 vaping-warning posts as well as 200 pro-vaping and 200 vaping-warning user accounts were randomly selected for hand coding. Furthermore, follower counts and media counts of the Instagram user accounts as well as the “like” counts and hashtags of the posts were compared between pro-vaping and vaping-warning groups.

**Results:**

There were more posts in the pro-vaping group (41,412 posts) than there were in the vaping-warning group (1539 posts). The majority of pro-vaping images were product display images (163/200, 81.5%), and the most popular image type in vaping-warning posts was educational (95/200, 47.5%). The highest proportion of pro-vaping user account type was vaping store (110/189, 58.1%), and the store account type had the highest mean number of posts (10.33 posts/account). The top 3 vaping-warning user account types were personal (79/155, 51%), vaping-warning community (37/155, 23.9%), and community (35/155, 22.6%), of which the vaping-warning community had the highest mean number of posts (3.68 posts/account). Pro-vaping user accounts had more followers (median 850) and media (median 232) than vaping-warning user accounts had (follower count: median 191; media count: 92). Pro-vaping posts had more “likes” (median 22) and hashtags (mean 20.39) than vaping-warning posts had (“like” count: median 12; hashtags: mean 7.16).

**Conclusions:**

Instagram is dominated by pro-vaping content, and pro-vaping posts and user accounts seem to have more user engagement than vaping-warning accounts have. These results highlight the importance of regulating e-cigarette posts on social media and the urgency of identifying effective communication content and message delivery methods with the public about the health effects of e-cigarettes to ameliorate the epidemic of vaping in youth.

## Introduction

### Background

Since the invention of electronic cigarettes (e-cigarettes) in 2003, e-cigarette use (vaping) has increased rapidly worldwide [1]. E-cigarettes have now become the most popular tobacco product among youth in the United States [2]. In the United States, nationwide, the percentages of middle school and high school students who use e-cigarettes increased from 0.6% and 1.5%, respectively, in 2011 to 10.5% and 27.5%, respectively, in 2019 [3,4]. While the long-term effects of vaping on health are not known, multiple studies have demonstrated that components in e-cigarette aerosols may cause severe health problems, including respiratory disorders, cardiovascular diseases, mental health problems, and possibly cancer [5-11]. In addition, nicotine, contained in e-cigarettes, can harm brain function and increase the risk of addiction to other substances [12].

“The science of distribution and determinants of information in an electronic medium, specifically the Internet, or in a population, with the ultimate aim to inform public health and public policy” is defined as infodemiology [13,14]. Analyzing how people communicate and share health-related information on the internet can provide valuable insights into population behavior [14]. Social media platforms, which have been widely used by e-cigarette companies and vape stores for marketing and promoting the sale of their products [15-17], are currently very popular in the United States. Online advertisements from e-cigarette companies and vape stores claim that e-cigarettes have multiple benefits, such as having smoking cessation functions, being more economical than smoking, being healthier than tobacco, and having multiple flavors as choices [18-20]. Studies showed that the exposure to prosubstance-related social media is related to higher substance consumption rates among young people [21,22]. Pokhrel et al [23] demonstrated the association between social media e-cigarette exposure and e-cigarette use beliefs and behavior. Furthermore, initial e-cigarette use has been found to be associated with subsequent cigarette smoking in youth [24-26].

Exposure to the visual imagery of vaping has been shown to act as a conditioned cue to evoke the desire for regular cigarette and e-cigarette use [27]. Instagram, one of the most popular social media platforms, has been regularly used by more than half of US youth and young adults [28,29]. Instagram provides a platform for individuals to upload pictures and videos using hashtags as keywords. Keyword searches allow people to explore the images linked to the hashtags, and therefore, interact with the user-generated content [16]. E-cigarette content is popular on Instagram, and advertising companies characterize e-cigarettes as novel devices [16,30,31]. While the e-cigarette–related posts on Instagram are dominated by vaping-promoting images, there is another voice claiming that vaping is potentially harmful. In 2014, the US Food and Drug Administration (FDA) proposed a deeming rule to regulate tobacco products, which included e-cigarettes [32]. In 2018, the FDA launched a comprehensive campaign in high schools and on social media, including Instagram, to warn youth about the potential risks of using e-cigarettes [33]. Although the FDA has developed various policies to regulate e-cigarettes and warn young people about potential health risks of e-cigarette use [32,33], the percentage of middle school and high school students who are e-cigarette users has been increasing [3,4].

### Objective

This study aimed to investigate current vaping-related content on Instagram by comparing user account activities and user engagement between pro-vaping and vaping-warning groups to improve public health awareness, and more importantly, to identify effective ways for future communication about the harm of e-cigarette use.

## Methods

### Hashtags on Instagram

We started with 2 groups of root keywords: vaping-related keywords and negative-root keywords. After reviewing hundreds of vaping-related Instagram posts and their hashtags, we determined the list of root vaping-related keywords. These keywords covered the essential pro-vaping products or behaviors on Instagram, such as *vape*, *vaping*, *ecig*, *juul*, *eliquid*, and *ejuice*. The negative-root keywords included *quit*, *stop*, *no*, *anti*, *bad*, *danger*, *against*, and *end*. The combinations of negative-root keywords and root vaping-related keywords created a new group of vaping-warning keywords (eg, *antivaping*, *endvape*). Each keyword was input into the search bar on Instagram, which showed a list of hashtags that were derived from that keyword, as well as the total number of posts that used the hashtag. In this way, the final list of pro-vaping hashtags and vaping-warning hashtags were generated and ranked by their total number of posts by October 19, 2019. Due to a significant imbalance in the numbers of hashtags as well as in the numbers of posts for each hashtag between the 2 groups, different cut-points were used to collect the pro-vaping (number of posts ≥3000) and vaping-warning (number of posts ≥3) hashtags. Popular hashtags from each group are presented in Multimedia Appendix 1.

### Data Collection

The posts were collected through Instagram’s application programming interface, using popular hashtags from both pro-vaping hashtag (*#ecig*, *#ecigarette*, *#ecigarettes*, *#ecigs*, *#ejuice*, *#electroniccigarette*, *#eliquid*, *#juul*, *#vape*, *#vapefam*, *#vapenation*, *#vapeon*, *#vapercon*, *#vapers*, *#vapes*, *#vaping*) and vaping-warning hashtag *(#novape*, *#novaping*, *#stopvaping*, *#dontvape*, *#antivaping*, *#quitvaping*, *#antivape*, *#stopjuuling*, *#dontvapeonthepizza*, *#escapethevape*) groups. Pro-vaping posts and vaping-warning posts were collected on November 18, 2019. Most posts used multiple hashtags, which resulted in duplicated posts when we collected data. Duplicated posts were excluded using the Instagram user ID and posting date. Random samples of posts and user accounts were selected for hand coding other features, including image type, attitude, and account type.

### Data Coding and Analysis

We first reviewed hundreds of posts and modified the coding scheme from a previous study [31] to develop our codebook. The codebook contained both metadata and other features that we created. Simple random sampling is a commonly used sample selection method that ensures the samples are representative of the population and that the statistics obtained from the sample are unbiased estimates of the population parameters [34]. Therefore, 200 posts each were randomly selected from both pro-vaping and vaping-warning data sets for hand coding using the simple random sampling method. This procedure was repeated twice to ensure that the 200 samples were representative of the whole data set. The proportions of each account type in each group were compared between 2 repeat samplings using the two-tailed chi-square test with a 5% level of significance.

For accounts, all the posts in each data set were grouped by user ID. Random user account samples (200 accounts) were selected from the Instagram account user IDs in each data set for hand coding. To increase the accuracy, the codebook was further revised during hand coding: All hand coding was performed independently by 2 authors, and the differences were resolved by discussions. The 2 reviewers’ agreement on classifying posts was very high (97.5%).

Instagram posts and user accounts were classified into pro-vaping (eg, promoting the use of e-cigarette–related products), vaping warning (eg, disagreeing with vaping behavior or emphasizing the potential health risks of vaping), neutral to vaping (eg, describing a fact related to e-cigarettes without clearly expressing an opinion), or not related to vaping (eg, having both image and caption not related to e-cigarettes, but using e-cigarette–related hashtags to target different groups of people). The visual and textual content were considered together to determine the attitude of each Instagram post [35]. The user account attitude was determined by browsing all the posts related to e-cigarettes on the user’s account page.

Type of image was categorized as (1) advertisement, for example, a picture displaying discount information of e-cigarette products; (2) catchphrase, for example, a picture emphasizing slogans such as “don’t juul in school” or “athletes don’t vape”; (3) product display, for example, a professional photo of an e-liquid container or e-cigarette device; (4) educational, for example, images that stated research results or facts about e-cigarettes; (5) events, for example, an image showing people attending a presentation or workshop related to e-cigarettes; (6) memes, for example, a picture created to deliver an e-cigarette–related message while being comedic; (7) news/notice, for example, image of a newspaper story or screen capture from TV of e-cigarette–related events; (8) vaping, for example, a person exhaling an aerosol; and (9) other, which included those not falling into any previously defined category. The image types were compared between the pro-vaping and vaping-warning posts.

Type of user account included (1) pro-vaping community, for example, a website claiming that e-cigarettes could benefit people; (2) personal, users who didn’t have an either commercial or professional affiliation; (3) sponsored vapor, an user who was sponsored by certain e-cigarette brands or stores and posted pictures of their products on Instagram; (4) store, for example, a grocery store selling e-cigarette products; (5) vaping store, a store that only sells e-cigarette products; (6) community, for example, a county uploading all local news, including e-cigarette–related information; (7) vaping-warning community, for example, a parent organization that is specifically against kids vaping; (8) business organization, for example, a law firm posting vaping-warning pictures to receive more vaping illness cases; and (9) influencer, users who have established credibility and a large number of followers.

The distribution of the follower count and the media count for each Instagram user account and the “like” count of each post were calculated, and the median values were compared between the pro-vaping group and the vaping-warning group using a two-tailed permutation test with a 5% level of significance. The mean number of hashtags and most frequently used hashtags in the 2 groups were calculated and compared using two-tailed two-sample *t* tests with a 5% level of significance.

## Results

### Characteristics of Pro-Vaping and Vaping-Warning Instagram Posts

While many pro-vaping hashtags had tens of millions of posts, the most popular vaping-warning hashtags in our list only had thousands of posts (Multimedia Appendix 1). Using pro-vaping and vaping-warning hashtags, we collected 41,412 unique pro-vaping posts and 1539 unique vaping-warning posts, of which, 200 pro-vaping and 200 vaping-warning posts were randomly selected for hand coding.

**Figure 1:**
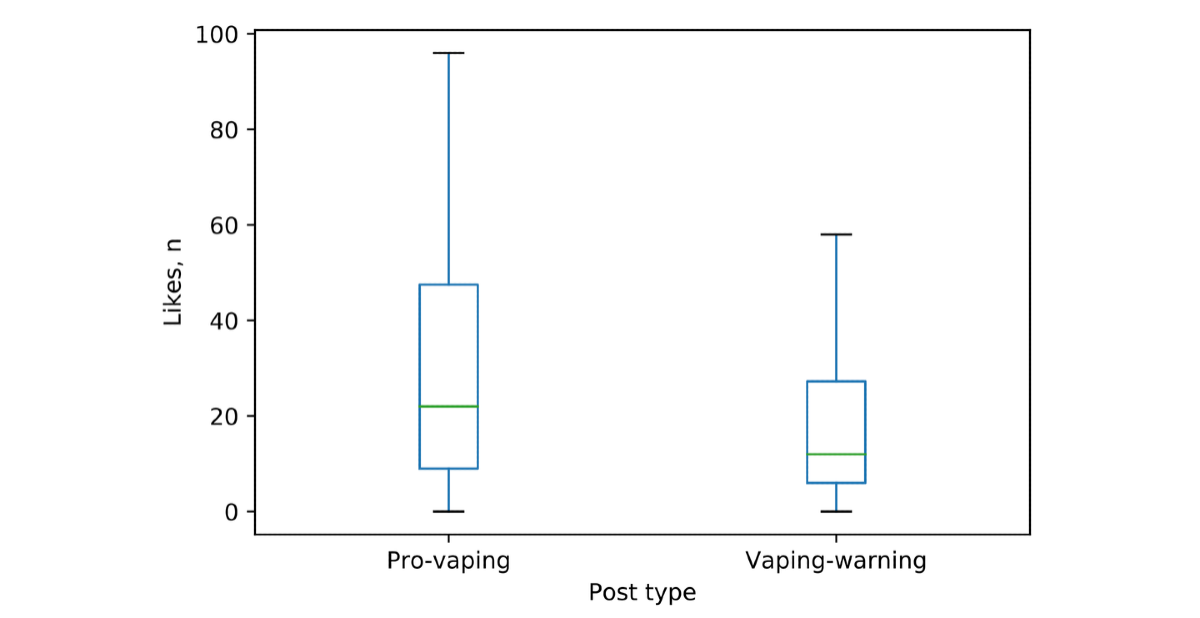
Number of "likes" for pro-vaping and vaping-warning posts.

**Figure 2:**
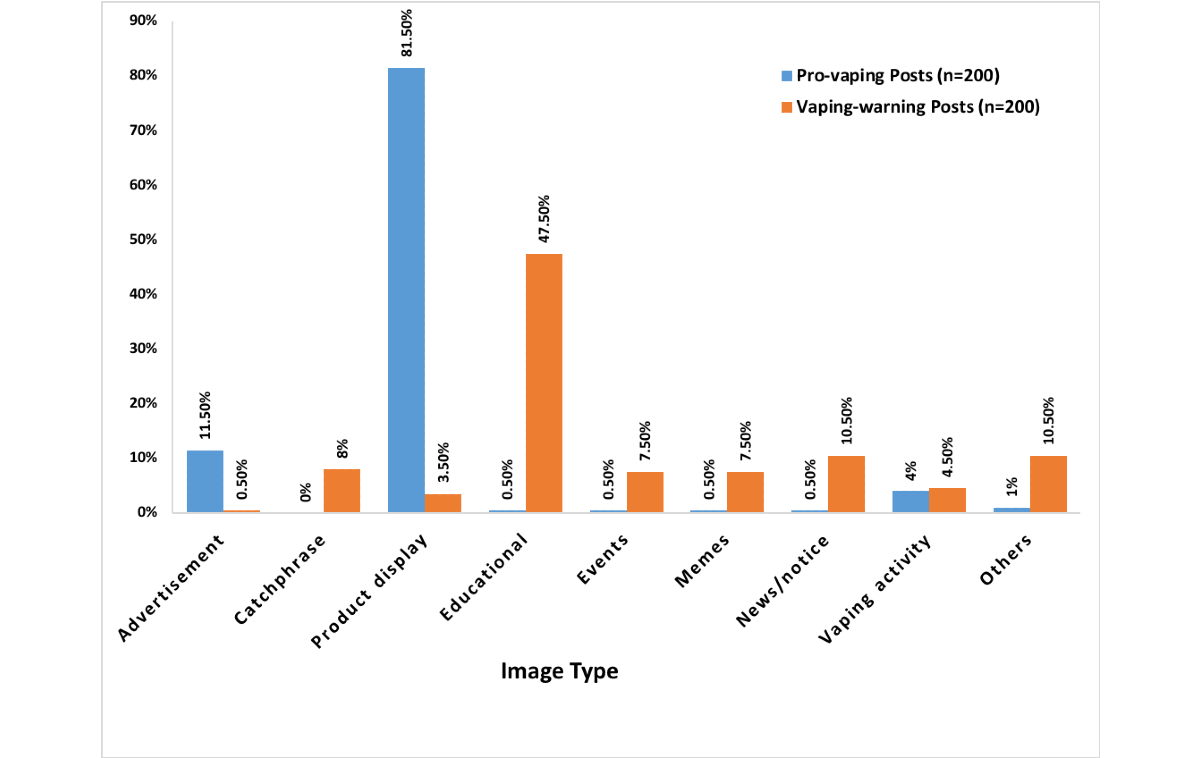
Image types of pro-vaping and vaping-warning posts.

Figure 1 displays the distribution of “like” counts of pro-vaping posts and vaping-warning posts. Pro-vaping posts (median 22) had more “likes” (*P*<.001) than vaping-warning posts had (median 12), although there were large within-group variations. The frequency distributions of image types were significantly different between pro-vaping posts and vaping-warning posts (*P<*.001) (Figure 2). Of the pro-vaping posts, product display–type images were the most common (163/200, 81.5%), followed by advertisement (23/200, 11.5%) and vaping activity (8/200, 4%). Among the vaping-warning posts, the most popular image type was educational (95/200, 47.5%), followed by news/notice (21/200, 10.5%), catchphrase (16/200, 8%), events (15/200, 7.5%), and memes (15/200, 7.5%). These analyses were repeated by randomly selecting another 200 pro-vaping and 200 vaping-warning posts. The percentages of image types in each group were consistent between the 2 repeat samplings (Multimedia Appendix 2 and Multimedia Appendix 3).

The number of hashtags for pro-vaping posts (mean 20) was significantly higher (*P<*.001) than that for vaping-warning posts (mean 7) (Multimedia Appendix 4). As one of the essential metrics on social media, the top 20 hashtags used for each group of posts are shown in Table 1. Each hashtag used in the pro-vaping posts was more frequently used compared to those used in the vaping-warning posts, which further demonstrated that pro-vaping posts generally used more hashtags. Some hashtags, such as *#vaping* and *#vape*, were widely used in both pro-vaping and vaping-warning posts.

**Table 1:** Top 20 hashtags in 200 pro-vaping and 200 vaping-warning posts.

Hashtags			Posts^a^, n (%)
**Pro-vaping**			
	#vape	116 (58.0)	
	#vapelife	85 (42.5)	
	#vapecommunity	78 (39.0)	
	#vapenation	76 (38.0)	
	#vapeon	74 (37.0)	
	#vaping	69 (34.5)	
	#vapeporn	68 (34.0)	
	#vapefam	68 (34.0)	
	#vapers	67 (33.5)	
	#eliquid	59 (29.5)	
	#vapedaily	55 (27.5)	
	#vapelyfe	51 (25.5)	
	#ecig	50 (25.0)	
	#vapestagram	49 (24.5)	
	#vapor	46 (23.0)	
	#ejuice	44 (22.0)	
	#vapeshop	43 (21.5)	
	#instavape	38 (19.0)	
	#vapelove	37 (18.5)	
**Vaping-warning**			
	#vaping	40 (20.0)	
	#vape	35 (17.5)	
	#stopvaping	29 (14.5)	
	#health	19 (9.5)	
	#dontvape	17 (8.5)	
	#juul	15 (7.5)	
	#nojuul	15 (7.5)	
	#novaping	14 (7.0)	
	#escapethevape	13 (6.5)	
	#vapingdangers	13 (6.5)	
	#tobaccofree	13 (6.5)	
	#teenvaping	12 (6.0)	
	#smokefree	12 (6.0)	
	#novapingcampaign	10 (5.0)	
	#breathealoha	10 (5.0)	
	#novape	10 (5.0)	
	#antivape	10 (5.0)	
	#endyouthvaping	9 (4.5)	
	#stopthevape	9 (4.5)	

^a^Hashtags could be found in more than one post.

### Characteristics of Pro-Vaping and Vaping-Warning Instagram User Accounts

**Figure 3:**
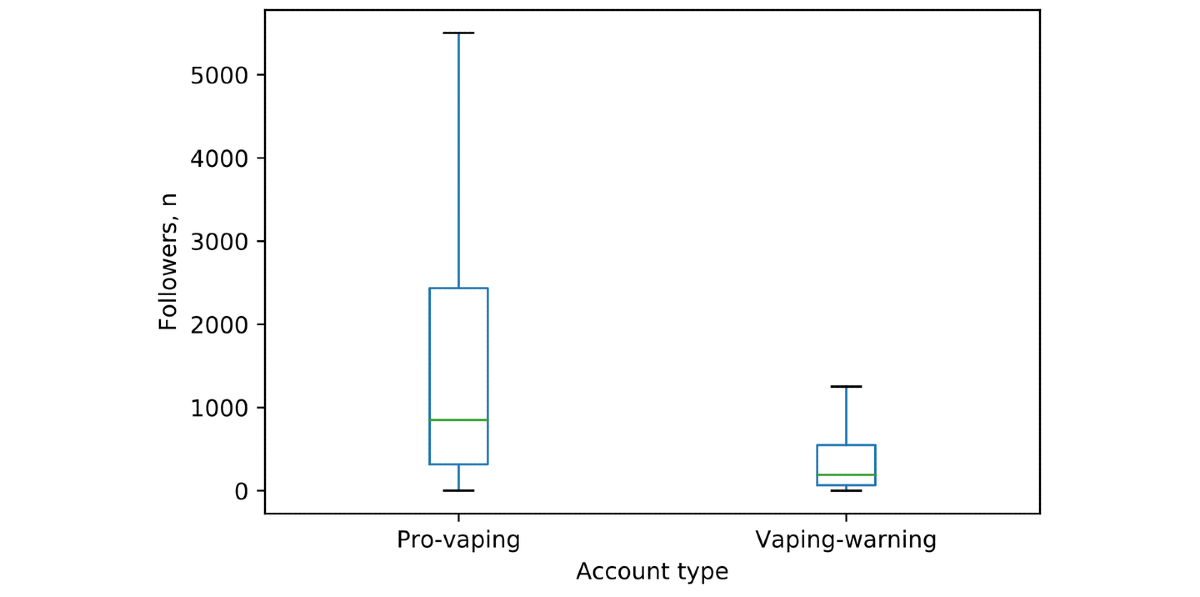
Follower counts of pro-vaping and vaping-warning Instagram accounts.

**Figure 4:**
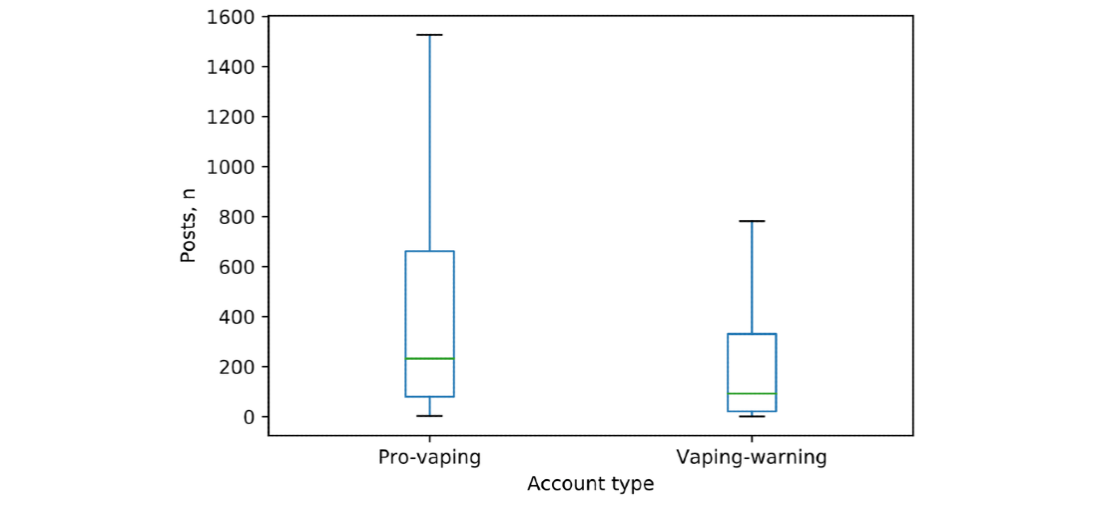
Media counts of pro-vaping and vaping-warning Instagram accounts.

The 41,412 pro-vaping posts identified in this study were posted by 18,074 unique Instagram user accounts, while the 1539 vaping-warning posts were posted by 712 unique user accounts. Out of the 200 randomly selected accounts from the pro-vaping group data set, 189 accounts (94.5%) were identified as pro-vaping user accounts. Out of the 200 randomly selected accounts in the vaping-warning data set, most were vaping-warning user accounts (155/200, 77.5%). Compared with those of the vaping-warning accounts (median 191, pro-vaping user accounts (median 850) had more followers (*P<*.001; Figure 3). The media (posts) count of the pro-vaping user accounts (median 232) was higher (*P<*.001) than that of the vaping-warning user accounts (median 92; Figure 4).

Table 2 summarizes the account types of pro-vaping accounts as well as pro-vaping posts collected from those user accounts. The most popular pro-vaping user account type was vaping store (110, 58.1%), which accounted for half of the posts (231/452, 51.1%); 18% of user accounts were personal (34/189), and 15.9% were sponsored vapor (30/189), which contributed 15.3% (69/452) and 10.3% (49/452) of posts, respectively. Only 3.2% (6/189) user accounts were pro-vaping communities, and only 4.8% (9/189) were stores; however, 20.6% of the pro-vaping posts (93/452) were from the store user accounts.

**Table 2:** Types of pro-vaping Instagram user accounts.

Account types		Accounts, n (%)	Pro-vaping posts, n (%)	Posts/account, mean
**Total**		**189 (100)**	**452 (100)**	**—^a^**
	Pro-vaping community	6 (3.2)	10 (2.2)	1.67
	Personal	34 (18)	69 (15.3)	2.03
	Sponsored vapor	30 (15.9)	49 (10.8)	1.63
	Store	9 (4.8)	93 (20.6)	10.33
	Vaping store	110 (58.1)	231 (51.1)	2.1

^a^Not reported.

Type and composition of vaping-warning user accounts and their posts is summarized in Table 3. The top 3 vaping-warning user account types were personal (79/155, 51%), vaping-warning community (37/155, 23.9%), and community (35/155, 22.6%), which posted 34% (99/291), 46.8% (136/291) and 17.9% (52/291) of the vaping-warning posts, respectively.

**Table 3:** The account types of the vaping-warning Instagram user accounts.

Account types		Accounts, n (%)	Vaping-warning posts, n (%)	Posts/account, mean
**Total**		**155 (100)**	**291 (100)**	**—^a^**
	Community	35 (22.6)	52 (17.9)	1.49
	Antivaping community	37 (23.9)	136 (46.8)	3.68
	Personal	79 (51)	99 (34)	1.25
	Influencer	1 (0.6)	1 (0.3)	1
	Business organization	3 (1.9)	3 (1)	1

^a^Not reported.

## Discussion

### Principal Findings

In this study, we showed that e-cigarette–related content on Instagram was highly imbalanced and dominated by pro-vaping posts. Most pro-vaping images were product display images, and vaping store was the most common user account type among pro-vaping user accounts. In contrast, the most popular image type of the vaping-warning posts was educational, and personal was the top vaping-warning account type among vaping-warning user accounts. In addition, pro-vaping user accounts had more followers and posts than those of vaping-warning users, and pro-vaping posts had more “likes” and hashtags than those of vaping-warning posts.

### Comparison With Prior Work

Vaping-related studies on social media mainly rely upon hashtags for collecting data. While some have directly used a few frequently appearing hashtags [16,17], others used hashtags by determining several root-term hashtags and finding co-occurring hashtags [30,31,36]. However, using these 2 methods, vaping-warning hashtags may be completely missing due to nonfrequent posting. Here, we developed a way to analyze the pro-vaping and vaping-warning hashtags on Instagram. The numbers of hashtags as well as the numbers of posts for each hashtag between the 2 groups were highly imbalanced (Multimedia Appendix 1). Even with different cut-points, the pro-vaping group still had more hashtags than vaping-warning group had. Considering the hashtags were used by e-cigarette companies as a strategy to promote their products [37], further study on how to efficiently use hashtags to improve the impact of vaping-warning campaigns is required.

On Instagram, each pro-vaping hashtag was found in more than 4000 posts, while each vaping-warning hashtag was only found in less than 100 posts. Further hand coding showed the majority of the posts and user accounts based on the pro-vaping hashtags were indeed pro-vaping. These results demonstrated that social media is dominated by vaping-promotion information, consistent with the findings of previous reports [38,39]. These analyses highlight the need for active public health engagement in communicating the harm of vaping on Instagram.

Figure 2 showed that the image types of pro-vaping posts were relatively consistent, while the image types of vaping-warning posts varied. Table 2 and Table 3 indicated different frequencies of posts by account types. Further analysis will be necessary to identify the vaping-warning account types and image types that have more impact in communication with the public, in order to help improve the efficiency in conveying the health risks of vaping to the public.

In this study, we showed that pro-vaping Instagram posts had a higher median “like” counts than those of vaping-warning posts (Figure 1). However, a person who “likes” a post does not necessarily support e-cigarette products or vaping-related content in the post. Most product-display images were professional pictures from vaping stores or well-designed pictures from sponsored photographers or models. Someone may “like” the post because of the design of the pictures (such as a beautiful view or a seductive model in the picture). In addition, our results showed that the number of followers and number of hashtags in the pro-vaping group were higher than those of the vaping-warning group. Followers [40] and hashtags [37] have been shown to be essential metrics on social media, which may help with the spread of information and increase the chance of getting “likes.” Although “likes” could be for multiple reasons, the high “like” count is always perceived as the behavior or content in the images being appropriate and accepted by society [41], which may cause more youth to start using e-cigarettes. Therefore, the policies for regulating the pro-vaping posts and approaches for improving user engagement with vaping-warning posts are in high demand.

Linnea et al [30] identified some hashtag communities (eg, *#vapefam*, *#vapecommunity*) used in the pro-vaping Instagram posts. Those users reinforced their identities through a folksonomy process [35,42]. We showed that there were some hashtag communities in the vaping-warning group (eg, *#atheletesdontvape*, *#parentsagainstvaping*). The self-identification within those communities may help spread warnings of potential health risks in the vaping-warning posts. Therefore, more of such folksonomy terms should be generated for different groups of people (eg, *#studentsdontvape* for middle and high school students, *#youthdontvape* for the youth) and adopted by more vaping-warning posts to effectively deliver vaping-warning information to diverse populations. Other than pro-vaping and vaping-warning hashtag communities, there were some hashtags widely used by both pro-vaping and vaping-warning posts. For example, *#vape* and *#vaping* were the top hashtags in both pro-vaping and vaping-warning groups, while most posts using these hashtags were pro-vaping. This phenomenon was mainly due to the fact that social media platforms are currently dominated by vaping brands. In addition, the high frequency of hashtags used in pro-vaping posts could contribute to this result. However, as more vaping-warning campaigns are launched, we should consider these vaping terms (eg, *#vape*, *#vaping*, *#ecig*, *#juul*) as general vaping-related hashtags without attitudes.

### Limitations

This study had several limitations. First, some popular hashtags are generated by describing vaping (eg, *#cloudchasing*, *#cloudchaser*, *#cloudchasers*) or vaping-warning (eg, *#choosecleanair*) content other than those directly derived from our root keywords and were missing in our analyses. Second, our data were collected using Instagram’s proprietary search algorithms, which may introduce inevitable and nontransparent biases to the investigation. Data-driven approaches will be used in future work. Third, we did not know the demographic information of Instagram users. Fourth, due to the low frequency of vaping-warning Instagram posts, we collected less than 2000 unique vaping-warning posts, while there were more than 40,000 pro-vaping posts collected. This imbalance may introduce some biases into our analysis. In the future, more vaping-warning posts will be collected to get a more balanced data set for detailed studies to identify critical features that have the potential to increase the impact of vaping-warning campaigns.

### Conclusions

This study reported and characterized the current imbalance in pro-vaping and vaping-warning content on Instagram, showing fewer posts and less user engagement of vaping-warning information. Our results highlighted the urgency to regulate social media on e-cigarette–related content. Vaping-warning posts could potentially use more hashtags or a better image designs for more user engagement and to deliver precise and proper e-cigarette–related information to the public, especially youth.

## Appendix

Multimedia Appendix 1 Pro-vaping and vaping-warning hashtags.

Multimedia Appendix 2 Comparison of the proportions of pro-vaping image types between 2 repeats.

Multimedia Appendix 3 Comparison of the proportions of vaping-warning image types between 2 repeats.

Multimedia Appendix 4 Average number of hashtags used by the pro-vaping and vaping-warning groups.

## Abbreviations

e-cigarette: electronic cigarette
FDA: US Food and Drug Administration
