# Supplementary figures and images for: Electronic Cigarette–Related Contents on Instagram: Observational Study and Exploratory Analysis
Source: JMIR Public Health Surveill. 2020 Nov 5;6(4):e21963. doi: 10.2196/21963 (PMC7677028; doi:10.2196/21963)

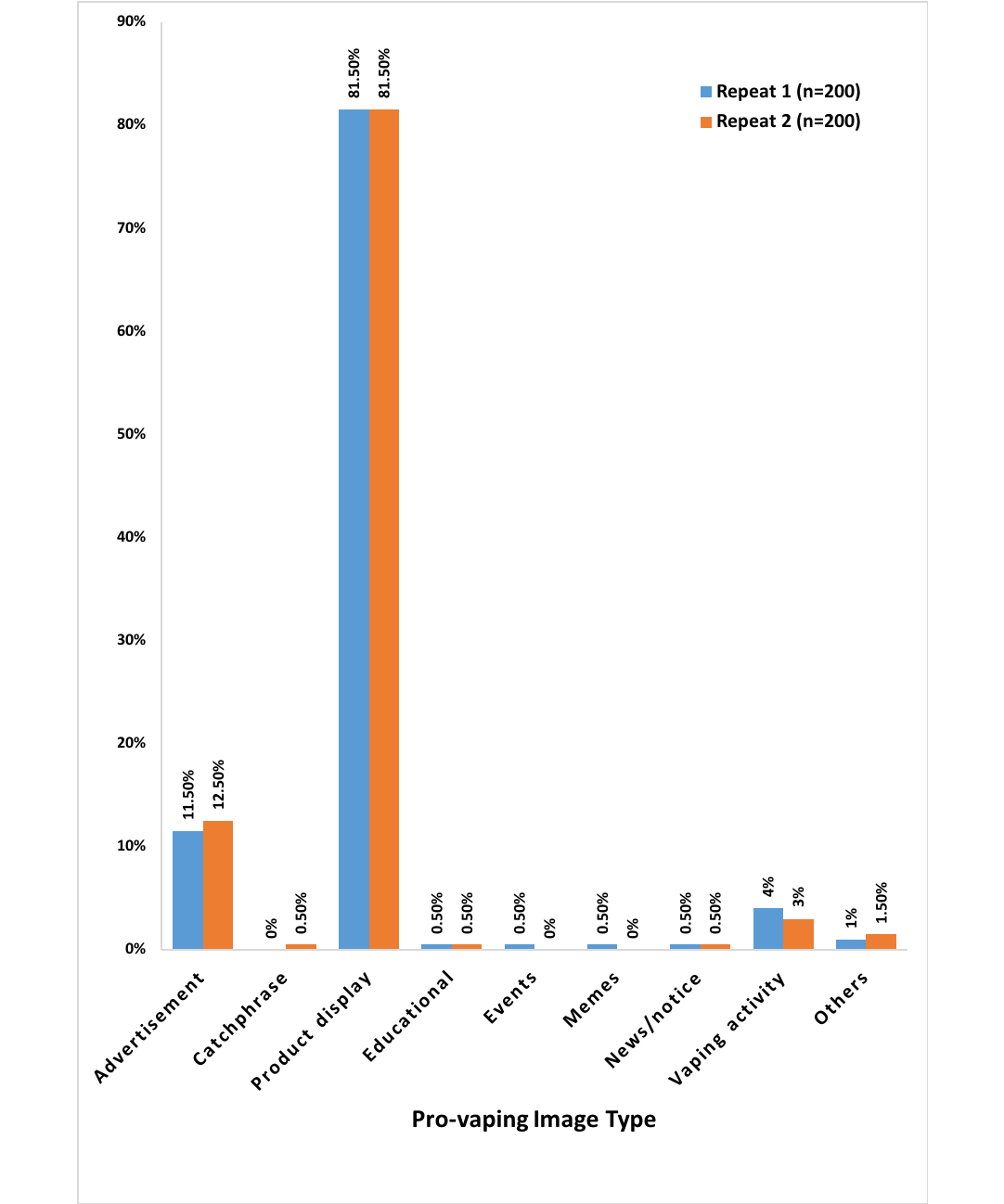

Supplement: Multimedia Appendix 2 [file publichealth_v6i4e21963_app2.png]

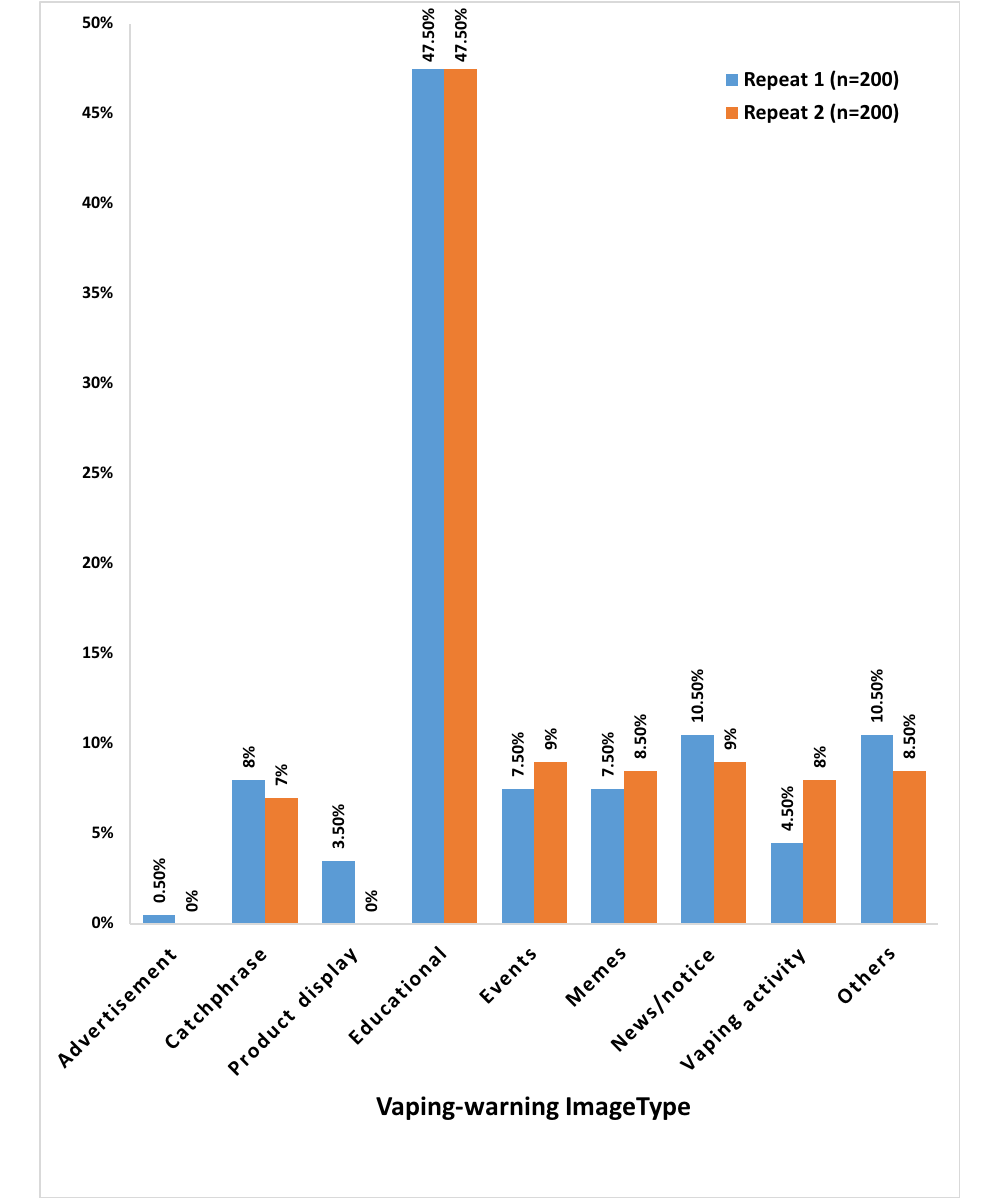

Supplement: Multimedia Appendix 3 [file publichealth_v6i4e21963_app3.png]

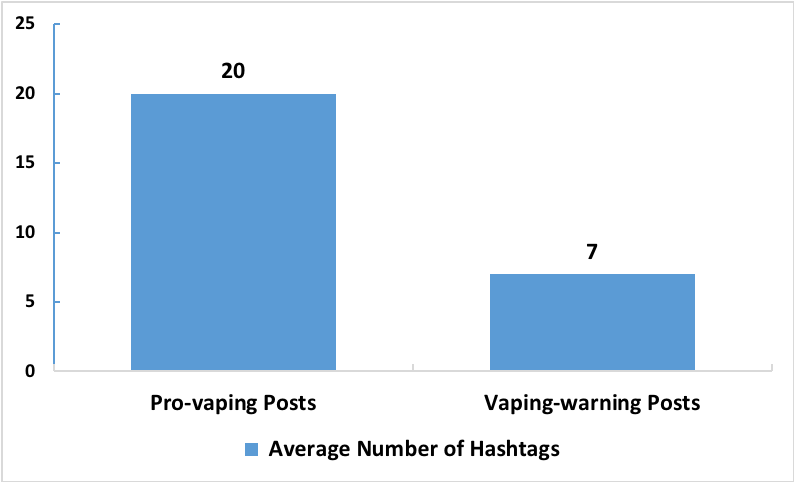

Supplement: Multimedia Appendix 4 [file publichealth_v6i4e21963_app4.png]
